# Variation in TAS2R receptor genes explains differential bitterness of two common antibiotics

**DOI:** 10.3389/fgene.2022.960154

**Published:** 2022-07-28

**Authors:** Alissa A. Nolden, John E. Hayes, Emma L. Feeney

**Affiliations:** ^1^ Department of Food Science, University of Massachusetts, Amherst, MA, United States; ^2^ Department of Food Science, The Pennsylvania State University, University Park, PA, United States; ^3^ Institute of Food and Health, University College Dublin, Dublin, Ireland

**Keywords:** T2Rs, bitter taste receptors, chloramphenicol, ofloxacin, propylthiouracil, prop, taste psychophysics, bitter drugs

## Abstract

For pharmaceuticals to deliver their full benefits with maximum efficacy, patients need to follow recommended dosing schedules, in terms of amount and frequency. Unfortunately, the aversive taste of many drugs, especially bitterness, can reduce patient compliance in oral liquid formulations. Given common genetic differences in bitter taste receptor genes (*TAS2Rs*), some individuals may be at increased risk for poor compliance due to heightened bitterness that becomes a barrier to proper use. Here we report on the sensory profile of two antibiotics, chloramphenicol and ofloxacin, investigating whether bitterness intensity associates with nominally functional *TAS2R* variants. Participants (*n* = 143) rated suprathreshold intensity on a general Labeled Magnitude Scale (gLMS) for chloramphenicol and ofloxacin; propylthiouracil (PROP) was included as a control, given robust prior associations with *TAS2R38* variants. The dominant sensation from chloramphenicol and ofloxacin was bitterness, falling just below “moderate” on a gLMS. *TAS2R38* diplotype associated with variable bitterness of chloramphenicol and PROP, but not ofloxacin. The bitterness of ofloxacin associated with a *TAS2R9* SNP (V187A). This pilot study provides novel evidence on differences in the bitterness from two antibiotics, which are associated with *TAS2R* variants. Improved understanding of individualized barriers to patient compliance, especially for oral formulations, can guide future efforts to optimize delivery systems for improved compliance.

## Introduction

Bitterness is innately aversive and generally associates with reduced liking and consumption of foods and beverages (Hayes, 2020; May-Wilson et al., 2022). While this relationship is widely studied in foods (e.g., vegetables, beer, dark chocolate) (Mattes, 1994; Keller et al., 2002; Dinehart et al., 2006) less quantitative evidence regarding aversive tastes and medication compliance exists for adults, but presumably, the same pattern exists (Shahiwala, 2011; Schiffman, 2015) especially given relevant aphorisms from diverse cultures. For example, there is a Chinese idiom (“liánɡ yào kǔ kǒu”) which claims, “good medicine tastes bitter,” while in English, Mary Poppins famously sang “a spoonful of sugar helps the medicine go down.” In a retrospective study with children, taste-related complaints were reported as the number one reason for rejection of liquid oral medication (Mennella et al., 2015). Collectively then, it seems reasonable to conclude that bitterness from medications may result in reduced compliance. Thus, we examined the sensory profile of two antibiotics, chloramphenicol and ofloxacin.

Despite long understood associations between bitterness and pharmacological activity [e.g., phlorizin from apple tree bark; see (Ehrenkranz et al., 2005)], quantitative data on the sensory profile of various medications is surprisingly sparse. Work by Schiffman and colleagues reported recognition thresholds of 42 medications, with all but one being bitter once the concentration was detectable (Schiffman et al., 2002; Schiffman, 2015). Although chloramphenicol was not assessed, the detection threshold of ofloxacin was estimated to be 0.38 mM and was reported to be bitter (Schiffman et al., 2002; Schiffman, 2015). Another study by Schiffman et al. (1999) reported the sensory profile for three protease inhibitors, ritonavir, indinavir, and saquinavir. Healthy controls and HIV-patients reported intensity ratings on a 7-point intensity scale for suprathreshold concentrations. Bitterness was the highest rated sensation, rated between “moderate” and “moderate strong” for all three drugs, with other sensations reported as having a “weak” intensity or lower, including metallic, medicinal, and astringent. Other drugs that have undergone formal sensory testing include the antiretroviral drug tenofovir alafenamide (TAF): when given to a small panel of healthy adults in a liquid formulation, the mean bitterness was rated near “moderate” on a general Labeled Magnitude Scale (gLMS), albeit with variable ratings ranging from “weak” to “very strong” (Schwiebert et al., 2021). Although individual differences were not explicitly investigated by those authors (perhaps due to the small sample size; *n* = 16), the wide range in intensity ratings suggests there may be substantial variability in the bitterness of this drug. Likewise, another antiretroviral cocktail (lopinavir/ritonavir) varied in bitterness ratings, with a downstream impact on hedonic ratings (Mennella et al., 2017). Two notable exceptions to the overall lack of quantitative sensory data on pharmaceuticals include the antimalarial drug quinine and the thyroid drug propylthiouracil (PROP)—in part because quinine is used outside a therapeutic context as a food ingredient (FEMA# 2,976) and PROP is commonly used as a probe of variation in taste (Smith and Davies, 1973; Lawless, 1980; Bartoshuk et al., 1992; Bartoshuk et al., 1994; Delwiche et al., 2001; Duffy et al., 2004; Chang et al., 2006; Hansen et al., 2006; Hayes et al., 2008; Wooding et al., 2010; Mennella et al., 2011; Feeney et al., 2014; Boxer and Garneau, 2015; Hayes et al., 2015; Nolden et al., 2020). Collectively, the limited data that exists suggests many commercial pharmaceuticals are perceived as bitter, and this bitterness may vary across individuals.

Differential bitterness has been repeatedly shown to associate with normal variation in bitter taste receptor genes (*TAS2Rs*). This gene family has 25 members which encode receptors (hT2Rs) tuned to a wide range of ligands (Meyerhof et al., 2010). Critically, the *TAS2Rs* are highly polymorphic relative to the rest of the human genome (Kim et al., 2005). Kim and colleagues were the first to identify a relationship between single nucleotide polymorphisms (SNPs) in the *PTC* gene (which was quickly renamed *TAS2R38),* and individual differences in perception of phenylthiocarbamide (PTC), a structurally similar chemical to PROP (Kim et al., 2003; Bufe et al., 2005; Kim et al., 2005). Subsequently, a robust relationship between *TAS2R38* genotypes and perception of PROP and PTC emerged e.g., (Duffy et al., 2004; Hayes et al., 2008; Allen et al., 2013). Such individual differences extend beyond PTC and PROP, with variation in the perception of diverse compounds that associates with polymorphisms in multiple *TAS2Rs* [e.g., (Allen et al., 2013; Allen et al., 2014; Risso et al., 2014; Hayes et al., 2015; Roura et al., 2015)].

Multiple studies have systematically investigated which hT2Rs are activated *in vitro* by specific bitter compounds, including drugs (Meyerhof et al., 2010; Thalmann et al., 2013; Levit et al., 2014) [see review (Clark et al., 2012)]. Such data initially suggested chloramphenicol is an agonist for six different hT2Rs, specifically, −1, −8, −10, −39, −43, and −46 (Meyerhof et al., 2010). Later, Thalmann and colleagues (2013) extended this set, by identifying hT2R41 as a potential receptor for chloramphenicol. In a series of functional experiments, they identified a genetic variant, L127, which stimulated an ∼10-fold reduction in response relative to cells that expressed the P127 variant of the receptor (Thalmann et al., 2013). The hT2R41 receptor appears to be narrowly tuned, as the only other ligand identified to date is the non-nutritive sweetener sucralose (Lossow et al., 2016), which is known to evoke bitter sensations at some concentrations (Antenucci and Hayes, 2015). The earlier work of Meyerhof and colleagues (2010) likely failed to identify chloramphenicol as an agonist of hT2R41 because the variant used in the original study was the non-functional variant, at least, according to Thalmann and colleagues (2013).

To our knowledge, only a single study has tested ofloxacin as a hT2R agonist: Dotson and colleagues (2008b) found hT2R9 responded to ofloxacin *in vitro* (along with two other drugs, procainamide and pirenzepine). They also reported functional variation in *TAS2R9* (V187A), with the A187 variant being the active form for all three drugs tested.

Collectively, these studies indicate that genetic differences in *TAS2Rs* are associated with bitterness perception of medications, with the implication that individuals expressing specific functional alleles for some *TAS2Rs* may have a greater risk for non-compliance. Mennella and colleagues (2015) provided evidence of this in children: in a retrospective interview, children with one or two copies of the functional *TAS2R38* variant (P49) reported greater rejection of liquid formulations of medications (type unspecified), relative to children with two copies of the non-functional variant (A49) of *TAS2R38*. To explore these types of relationships, we 1) obtained sensory profiles of two antibiotics (chloramphenicol and ofloxacin) in sample of nominally healthy adults, and 2) tested to see if variation in bitterness ratings could be explained by functional polymorphisms in *TAS2Rs*. We also 3) included PROP as a positive control, given the robust association between PROP bitterness and *TAS2R38* diplotype (e.g., Nolden et al., 2020).

## Methods

### Overview

This study consisted of two 1-h lab visits, scheduled 1 week apart. All sessions took place in the Sensory Evaluation Center at Penn State between Augest and October 2013. During the first session, each participant met with a researcher who obtained informed consent and provided an overview of the study objectives. This was followed by the collection of a salivary DNA sample (detailed below) and anthropometric measures. The tasting procedure for Sessions 1 and 2 was similar: participants entered an individual testing booth and received written instructions on a computer that described the order of tasks. Prior to tasting any samples, participants completed an orientation on scale usage for the suprathreshold intensity ratings (detailed below). In both Sessions 1 and 2, they tasted and rated a total of 45 stimuli. Here, we are focused on the ratings of chloramphenicol, ofloxacin, and propylthiouracil (PROP), which were each collected in duplicate. All data were collected using Compusense five (version 5.2, Guelph ONT). The study was performed in accordance with the Declaration of Helsinki and the protocol was approved by professional staff in the Penn State Office of Research Projections (Protocol #33176).

### Participants

Adults were recruited from the Pennsylvania State University campus and the surrounding community. Interested individuals were screened using an online survey to determine if they met the inclusion criteria: between 18 and 45 years old, not pregnant or breastfeeding, nonsmoker, no tongue, cheek, or lip piercings, no history of chronic pain, no known smell or taste defect, no hyperactive thyroid, and willingness to provide a salivary DNA sample, and self-identify as being of European Ancestry. This was done to increase sample homogeneity, given the demographics of the local region, and well-known differences in *TAS2R* allele frequencies across ancestral groups e.g., (Soranzo et al., 2005). A total of 154 participants consented, and 149 completed both tasting sessions. Six of the 149 individuals were excluded from analysis due to missing data, resulting in a final dataset of 143 participants (101 females). The mean age was 25.5 years (SD ± 6.5), and all participants reported European Ancestry, per screening criteria.

### Stimuli and sampling protocol

The present analysis investigates the psychophysical response to chloramphenicol and ofloxacin, using direct scaling. PROP was included as positive control and as a comparative bitter stimulus. Other taste stimuli presented during the lab visits were not analyzed here and will be detailed elsewhere. Specific stimuli used for this analysis include 0.1 mM chloramphenicol, 1 mM ofloxacin, and 3.2 mM PROP, each presented in duplicate, as 10 ml aliquots in clear plastic medicine cups. Participants were instructed to place the whole sample in their mouth, swish for 3 s and then expectorate before rating. To minimize risk to participants, all stimuli were expectorated without swallowing to reduce exposure to pharmaceutically active stimuli. Further, doses used here were explicitly chosen to be far below therapeutic levels. For example, a single 10 ml aliquot of 0.1 mM chloramphenicol would contain 323 μg; comparatively, the daily therapeutic dose for a 50 kg woman would be 2,500,000 μg (i.e., a safety factor of ∼3.9 orders of magnitude). All solutions were prepared with reverse osmosis water and stored refrigerated for no more than 1 week prior to use, and all samples were brought to room temperature prior to tasting. Samples were labeled with three-digit blinding codes. Presentation order was counterbalanced with a Williams Design (Williams, 1949). A minimum interstimulus interval of 30 s was enforced between stimuli.

### Psychophysical scaling

Participants rated the intensity of all sampled stimuli on a general labeled magnitude scale (gLMS) (Snyder et al., 2004), with separate ratings for sweetness, bitterness, sourness, saltiness, burning/stinging, and tingling/pricking. The gLMS ranges from 0 (“no sensation”) to 100 (“the strongest imaginable sensation of any kind”), with descriptors placed at 1.4 (“barely detectable”), 6 (“weak”), 17 (“moderate”), 35 (“strong”), and 51 (“very strong”). Before tasting any stimuli, participants received written instructions on the use of the scale, including statements to not let the liking or disliking of a sample influence their intensity rating, and to use the whole length of the scale rather than just clicking on the semantic descriptors. To help participants practice using the scale, participants rated 15 imagined or remembered sensations, including both oral and non-oral sensations (Hayes et al., 2013). This procedure was intended to promote the use of the scale in a generalized context that was not limited to oral sensations and to ensure participants use the scale as instructed.

### Genetic analysis

DNA was collected from saliva using Oragene collection kits, according to the manufacturer’s instructions (Genotek Inc.). Individuals were genotyped for SNPs, Ala49Pro (A49P, rs713598) and Ala262Val (A262V, rs1726866) in *TAS2R38* located on chromosome 7, and Val187Ala (V187A, rs3741845) in *TAS2R9* located on chromosome 12 using Sequenom Mass ARRAY technology (Sequenom). MassARRAY software (Sequenom) automatically assigned genotypes and was subsequently independently inspected by two technicians. For the Ile296Val (I296V, rs10246939) SNP in *TAS2R38*, genotypes were determined by Taqman assay. For all assays, 15% of samples were rerun to ensure reliability. All primers were purchased from Integrated DNA technologies. The three SNPs in *TAS2R38* exhibit high linkage disequilibrium in European populations, resulting in two common genotypes being AVI and PAV. Two individuals were missing the genotype for A262V, and 9 others were not common genotypes (i.e., not AVI or PAV).

### Statistical analysis

Because ratings for each stimulus were collected in duplicate, we first calculated stimulus specific means for each participant. These means were then log transformed [e.g., (Lapis et al., 2014)]—this was done by adding the smallest possible non-zero rating (0.5 units on a gLMS) to all values (to eliminate any zeros) and then taking the log10 of each. All statistical analyses used these log-transformed values. Pearson correlations were calculated to test for any association between phenotypic values (i.e., bitterness ratings) and the three stimuli. Relationships between bitterness ratings and genetic variants were tested *via* analysis of variance (ANOVA); pairwise comparisons were adjusted for multiple comparisons using the Tukey-Kramer method. All analyses were performed in RStudio (version 2022.02.2 + 485).

## Results

### Comparison of the bitterness ratings of PROP, chloramphenicol, and ofloxacin

The dominant sensation of chloramphenicol and ofloxacin was bitterness. The non-normalized intensity ratings for both chloramphenicol and ofloxacin fell near “moderate” on a gLMS (16.4 ± 14.0 and 14.0 ± 13.5, respectively). For comparison, the grand mean of PROP bitterness (across all participants) was rated near “strong” (33.1 ± 21.8). On average, the next most intense sensation was drying, rated near “weak” for both chloramphenicol (5.8 ± 8.8) and ofloxacin (4.5 ± 6.4). For both chloramphenicol and ofloxacin, all other sensations had a mean rating of 2.4 or less on a 100-point gLMS, consistent with an interpretation that these stimuli are bitter without other meaningful side-tastes/sensations (radar plots of the various attribute ratings are shown in Supplementary Figure S1). Accordingly, we focus exclusively on bitterness ratings for the remainder of the manuscript.

**FIGURE 1:**
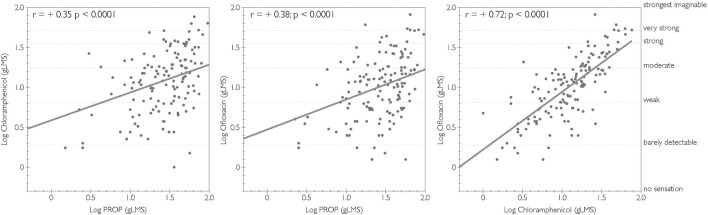
Correlations of logged bitterness ratings between chloramphenicol, ofloxacin, and PROP. Adjectives on the right y-axis refer to semantic labels on a gLMS.

Pearson correlations revealed significant associations between the bitterness of chloramphenicol, ofloxacin, and PROP, but the strength of this association varied, as shown in Figure 1. While there was a modest significant correlation between PROP and both chloramphenicol and ofloxacin, the strongest correlation was observed between chloramphenicol and ofloxacin.

### The bitterness of chloramphenicol is associated with *TAS2R38* diplotype

**FIGURE 2:**
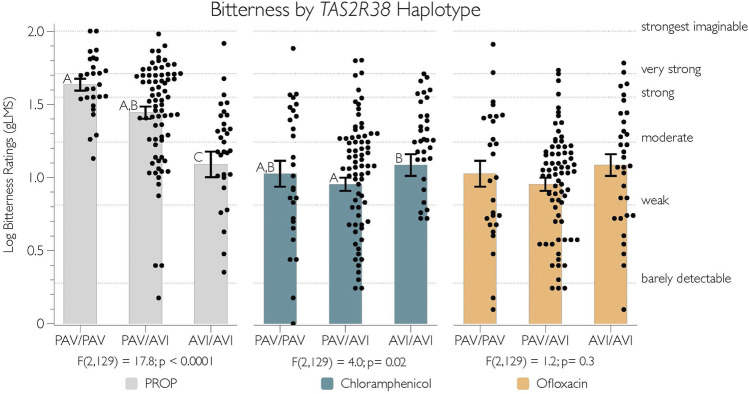
The logged bitterness of PROP and chloramphenicol is associated with *TAS2R38* diplotype. Adjectives on the right y-axis refer to semantic labels on a gLMS.10.3 Figure 3

We tested for a relationship between *TAS2R38* diplotype (PAV/PAV *n* = 27; PAV/AVI, *n* = 73; AVI/AVI, *n* = 32) and bitterness ratings for PROP, chloramphenicol, and ofloxacin by conducting an individual ANOVA for each compound. These results are summarized in Figure 2. As expected, there was a significant relationship between *TAS2R38* diplotype and PROP bitterness [F(2,129) = 17.8; *p* < 0.0001] (Figure 2, grey bars). AVI homozygotes reported lower bitterness of PROP than heterozygotes and AVI homozygotes (Tukey’s *p* < 0.0001). We observed weak evidence that PAV homozygotes may have found PROP to be more bitter than did heterozygotes (*p* = 0.06).

Unexpectedly, the bitterness of chloramphenicol was also varied by *TAS2R38* diplotype [F(2,129) = 4.0; *p* = 0.02] (Figure 2, blue bars). However, this may be a false positive, as the most bitterness was reported by AVI homozygotes, and it was only significantly different from the heterozygotes (*p* = 0.02), with no other differences observed between groups (*p*-values all > 0.08). As expected, the bitterness of ofloxacin was not associated with *TAS2R38* diplotype (*p* = 0.3) (Figure 2, yellow bars)

### The bitterness of ofloxacin is associated with V187A variant in *TAS2R9*

**FIGURE 3:**
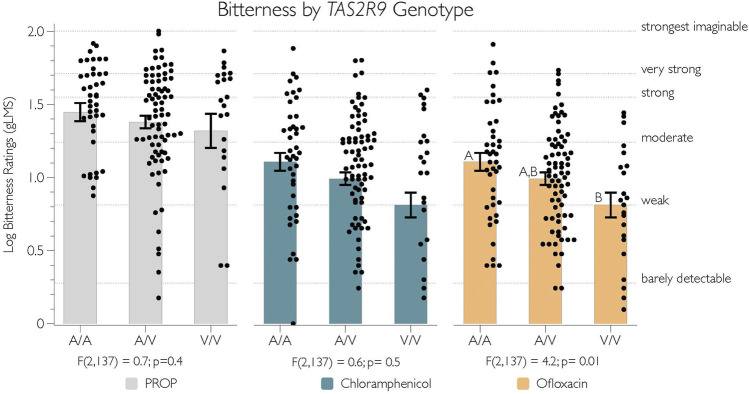
The logged bitterness of ofloxacin is associated with V187A SNP in *TAS2R9*. Adjectives on the right y-axis refer to semantic labels on a gLMS.

The putatively functional variant in *TAS2R9* was tested for a relationship with the bitterness of PROP, chloramphenicol, and ofloxacin, as shown in Figure 3. As expected, there was no evidence that the bitterness of PROP and chloramphenicol differed by the V187A polymorphism (*p*-values > 0.4). However, consistent with Dotson and colleagues (2008a), we did see evidence that this allele associates with variation in bitterness from ofloxacin [F(2,137) = 4.2; *p* = 0.01]. Specifically, we found that A187 homozygotes (*n* = 42) rated significantly more bitterness from 1 mM ofloxacin than V187 homozygotes (*n* = 22) (*p* = 0.01), with no differences between the heterozygote group (*n* = 76) and homozygote groups (*p*-values all > 0.27) (Figure 3, yellow bars).

## Discussion

Our working hypothesis is that there are individual differences in the bitterness from medications which are mediated by genetic variability in *TAS2Rs*; we reasoned such variation may put some individuals at greater risk for lower compliance due to heightened bitterness, so we asked whether such differences might manifest phenotypically. Here, we report the first systematic investigation of the bitterness of chloramphenicol and ofloxacin. Our data suggest individual differences in the bitterness perception extend to antibiotics, and these differences are systematically related to *TAS2R* variants previously shown to be functional. We also observed a positive correlation between the bitterness ratings of PROP and both chloramphenicol and ofloxacin, with the strongest correlation reported between chloramphenicol and ofloxacin. While PROP bitterness is associated with genetic polymorphisms in *TAS2R38* (Duffy et al., 2004; Kim et al., 2005), not all variability in PROP is explained by *TAS2R38* (Hayes et al., 2008). This is demonstrated by the relationship between ofloxacin and PROP but not with the *TAS2R38* diplotype. This finding aligns with prior work showing bitterness (Nolden et al., 2020) and sweetness (Allen et al., 2013) are each associated with PROP phenotype but not with *TAS2R38* genotype. That is, PROP bitterness is a phenotype that appears to confound narrowly tuned genetic variation (from *TAS2R38*) with a broader factor that predicts differences in orosensation.

Present data reinforce the consistent findings that *TAS2R38* variants can explain individual variability in the bitterness perception of PROP. Here, *TAS2R38* genotypes were included primarily as a positive control due to the many studies demonstrating a robust relationship with PROP bitterness (Duffy et al., 2004; Kim et al., 2005; Hayes et al., 2008; Wooding et al., 2010; Allen et al., 2013; Garneau et al., 2014; Nolden et al., 2020), which were confirmed yet again in the present study (Figure 2). Also, we note that Figure 2 indicates that some AVI homozygotes still reported moderate to strong bitterness from PROP, despite having two copies of the AVI variant of hT2R38, which is thought to be non-functional. Data in Figure 2 are consistent with the “second receptor” hypothesis, wherein another hT2R receptor that ligates PROP may recover function in some individuals who carry two copies of the non-functional *TAS2R38* allele see discussion in (Hayes et al., 2008; Nolden et al., 2020).

The observed relationship between chloramphenicol bitterness *in vitro* and *TAS2R38* diplotype was wholly unexpected, since prior *in-vivo* data from heterologous expression systems suggest hT2R38 is not activated by chloramphenicol (Meyerhof et al., 2010). To the best of our knowledge, we are unaware of any prior investigation into whether ofloxacin activates hT2R38, and in the present study, ofloxacin bitterness did not associate with *TAS2R38* diplotype. The AVI homozygotes tested here reported the highest bitterness, which would suggest that the AVI genotype might be the functional variant. We suspect this might be an artifact arising from linkage disequilibrium (LD) with a functional variant in another nearby *TAS2R* gene. This type of association has been reported previously for *TAS2R19* and *TAS2R31*, where polymorphisms in both predict responses to quinine and grapefruit, but only *TAS2R31* is thought to be causal see discussion in (Hayes et al., 2015). Here, we speculate one or more *TAS2R38* SNPs may be in LD with an unmeasured and functional variant of another *TAS2R*. For example, Thalmann and colleagues (2013) reported the P127L variant of hT2R41 corresponded to differential activation by chloramphenicol *in-vitro*, but we cannot test this directly, as we do not have data on *TAS2R41* variation in our participants. Thus, more work is needed to confirm the potential relationship between the bitterness of chloramphenicol and variability in *TAS2R38* and/or *TAS2R41*, as well as exploring possible relationships with other *TAS2Rs*, since prior data suggests chloramphenicol activates 6 other hT2Rs *in-vitro*. Alternatively, we cannot rule out the possibility that current data are a Type I error or that prior *in-vitro* data reflect a Type II error.

Separately, we observed as significant the relationship between the *TAS2R9* V187A polymorphism and the bitterness from ofloxacin, with the A187 form being the nominally-functional variant. These data align with prior work suggesting A187 responds to ofloxacin *in vitro* with reduced activation for V187 variant (Dotson et al., 2008). Previously, we reported V187A corresponds to variability in the bitterness from acesulfame-K (a widely used non-nutritive sweetener); however, in those data, V187 was associated with greater bitterness (Allen et al., 2013). More work is needed to clarify this discrepancy. Notably, Meyerhof and colleagues (2010) acknowledged that in their 2010 study, the hT2R9 amino acid sequence included the non-functional variant (V187); further, ofloxacin (nor procainamide or pirenzepine, which were used by Dotson et al. (2008)) was not included in the experiments by Meyerhof’s group (2010).

A substantial limitation of the present study is that our participants were not genotyped for the nominally functional variant of *TAS2R41* previously shown to be activated by chloramphenicol in a heterologous expression system (Thalmann et al., 2013). Still, we can speculate that the relationship observed here between the *TAS2R38* diplotype and the bitterness of chloramphenicol arises from LD between *TAS2R38* and *TAS2R41* variants. However, Meyerhoff and colleagues (2010) also identified 6 other hT2Rs that respond to chloramphenicol. It is possible that genetic variants in one or more of these receptors may be in LD with *TAS2R38* SNPs. Another limitation of this study is the concentrations of chloramphenicol and ofloxacin that were used—specifically, the concentrations used were well below the therapeutic dose, to minimize potential risk to the participants that come with sampling pharmacologically active stimuli. Further, all stimuli were expectorated and not swallowed, to further reduce exposure and thus participant risk; however, this also means we might not have adequately stimulated receptors in the throat see (Bennett and Hayes, 2012; Running and Hayes, 2017). While the present data provide new insight into the bitterness arising from these two antibiotics, we cannot definitively predict what the sensory profile of these medications might be closer to their therapeutic dose. Moreover, it is unknown whether genetic variants would still be associated with the bitterness perception at higher concentrations and if this would translate to differential compliance. Dosing form matters as well, as tablets or capsules help reduce aversive sensations that may be present in liquid oral formulations. If perceived bitterness of therapeutics negatively and meaningfully impacts compliance, greater consideration for sensations arising from pharmaceuticals and their formulation seems warranted. It is plausible that in the future, patients could receive personalized formulations of medications, informed from their genetic profile for *TAS2Rs* or other taste receptors.

Here, we illustrate individual differences in bitterness from two antibiotic medications and show that such variation may be due to genetic variability in *TAS2Rs*. Strategies for minimizing bitterness perception should consider individual differences driven by genetic variants in *TAS2Rs*. This study also highlights the importance of considering a personalized approach to screening medications and identifying bitter blockers to increase acceptance of medications and drug formulations more effectively. Future studies should investigate personalized medications not only for their efficacy but also for their taste profile, minimizing the aversive sensations such as bitterness, that ultimately may lead to improved compliance.

## Data Availability

The dataset presented in this study can be found in an online repository. The names of the repository/repositories and accession number(s) can be found in the article/Supplementary Material.

## Supplementary material

**Supplementary Figure S1** | Radar plot of intensity ratings (gLMS) for chloramphenicol and ofloxicin. Reported mean ratings are unlogged.
