# Supplementary figures and images for: Variation in TAS2R receptor genes explains differential bitterness of two common antibiotics
Source: Front Genet. 2022 Jul 28;13:960154. doi: 10.3389/fgene.2022.960154 (PMC9366911; doi:10.3389/fgene.2022.960154)

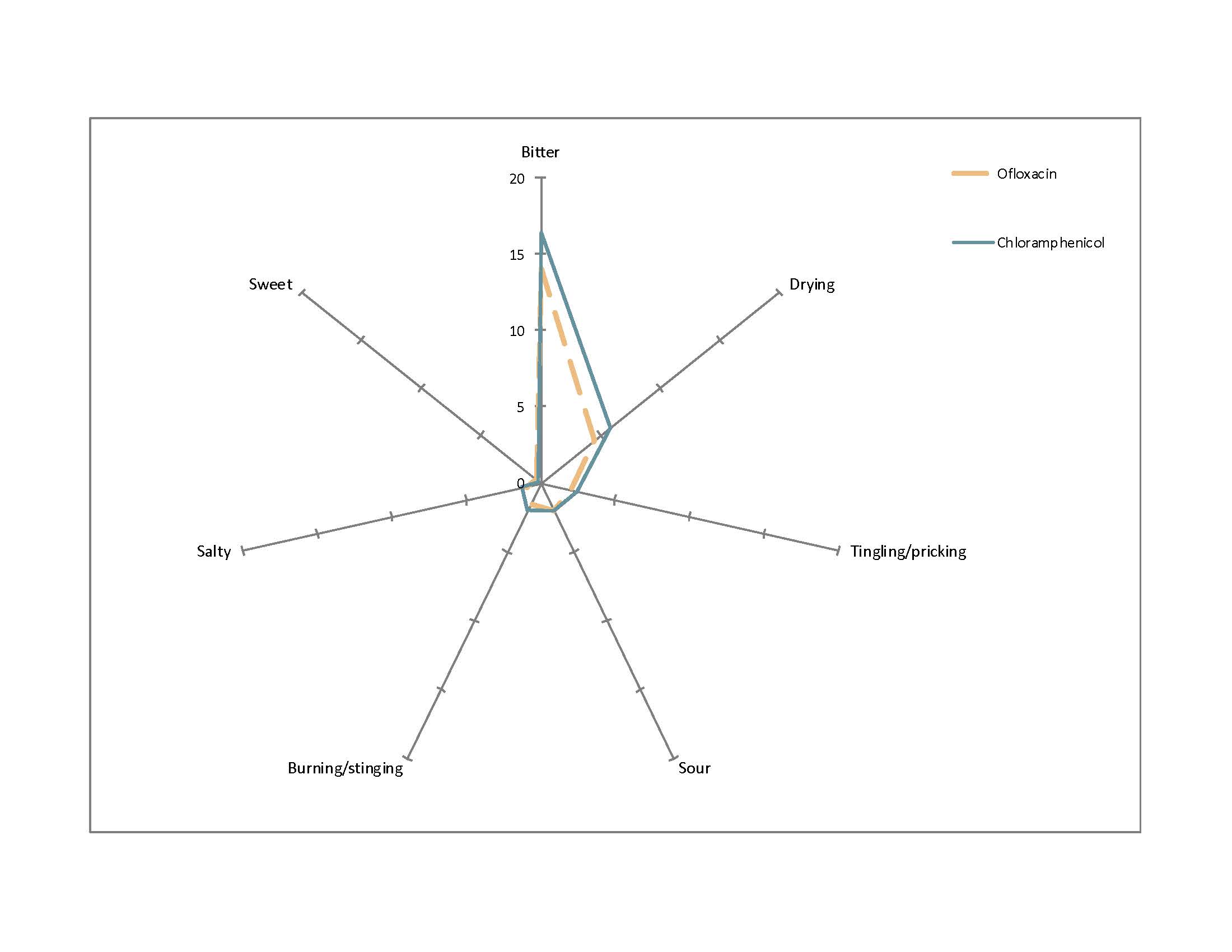

Supplement: Supplementary file 1 [file Image1.jpeg]
